# Intensity-modulated radiotherapy versus three-dimensional conformal radiotherapy for stage I-II natural killer/T-cell lymphoma nasal type: dosimetric and clinical results

**DOI:** 10.1186/1748-717X-8-152

**Published:** 2013-06-25

**Authors:** Qianwen Shen, Xuejun Ma, Weigang Hu, Lanfei Chen, Juan Huang, Ye Guo

**Affiliations:** 1 Department of Radiation Oncology, Fudan University Shanghai Cancer Center, Shanghai, China; 2 Department of Medical Oncology, Fudan University Shanghai Cancer Center, Shanghai, China; 3 Department of Oncology, Shanghai Medical College, Fudan University, Shanghai, China

**Keywords:** Nature killer (NK)/T-cell lymphoma, Three-dimensional conformal radiotherapy (3DCRT), Intensity-modulated radiotherapy (IMRT), Treatment outcome

## Abstract

**Background:**

This study was to compare radiotherapy treatment planning and treatment outcomes following three-dimensional conformal radiotherapy (3DCRT) and intensity-modulated radiotherapy (IMRT) in stage I-II natural killer (NK)/T-cell lymphoma.

**Methods:**

The cases of 94 patients with stage I-II NK/T-cell lymphoma, nasal type in the upper aerodigestive tract who treated between May 2005 and Dec 2008 were reviewed. These patients received radiotherapy with or without induction chemotherapy. Definitive radiotherapy was conducted using 3DCRT in 47 patients and IMRT in the other 47 patients with a regional field and a total dose of 50 Gy. Dosimetric pmeters of radiation treatment plans, local control probability (LCP), overall survival (OS), and toxicities were analyzed and compared between 3DCRT and IMRT.

**Results:**

From the dosimetric analysis, IMRT demonstrated significantly better dose coverage and homogeneity than 3DCRT. However, after a median follow-up of 46 months, IMRT was not associated with improvements in 4y-OS (80.9% for 3DCRT vs. 82.7% for IMRT, *p*=0.87) or 4y-LCP (86.3% for 3DCRT vs. 88.9% for IMR *p*=0.85). Of the 18 patients who received cervical lymph node irradiation, those in the IMRT group received a lower mean parotid dose. Furthermore, at-risk organs were strictly kept within the safe dose range in both groups, and no severe late toxicity was observed.

**Conclusions:**

IMRT provided better dose coverage than 3DCRT, although it failed to provide LCP and OS benefits. Definitive radiotherapy with a regional field and a total dose of 50 Gy is efficient and safe for NK/T-cell lymphoma using either IMRT or 3DCRT. However, IMRT may have the potential to reduce parotid gland hypofunction following cervical irradiation.

## 

Extranodal nature killer (NK)/T-cell lymphoma, nasal type is an aggressive type of lymphoma [1], which has been formally identified and classified as a distinct clinicopathologic entity according to the WHO classification in 2001. Clinically, this type of lymphoma is characterized by the presence of destructive disease in mid-facial structures. Although rarely diagnosed in Western countries, this subtype is prevalent in certain areas of Central America, South America, and East Asia [2]. The nasal cavity is the most commonly involved site of primary disease. Tumors with a same phenotype and genotype that are located in a variety of extranodal sites other than the nasal cavity are referred to as nasal-type tumors [3].

Although approximately 80% of cases diagnosed with localized disease are of stage I-II [4], treatment outcomes remain unsatisfying. In comparison to other subtypes of NHL, NK/T-cell lymphoma is relatively resistant to anthracycline-based chemotherapy [2,4-6], although it demonstrates a rapid and dramatic response to radiotherapy [4,5,7-10]. As radiotherapy is regarded as the primary therapy for stage I-II NK/T-cell lymphoma, the construction of feasible and optimal patterns of radiotherapy is critical. Nevertheless, the low incidence of NK/T-cell lymphoma and its confusing nomenclature has made it difficult to perform large-sample, prospective, randomized clinical trials. As a result, optimal radiotherapeutic models, including radiation field, dose and treatment plans, have not been properly established.

Though highly conformal radiation techniques have been widely studied in head-and-neck cancer, previous investigation of NK/T lymphoma treated with IMRT or 3DCRT is rare. The only study was reported by Wang H, et al. [11]. They got a favorable prognosis (the 2-year LRC, OS rate was 93% and 78%, respectively) and mild toxicity in 42 nasal NK/T-cell lymphoma cases with IMRT techniques. In comparison to IMRT, 3DCRT has poor conformal planning treatment volume (PTV) coverage, organ at risk (OAR) avoidance, and higher toxicity rates for a variety of cancers [12-15]. In contrast, with the use of complex computerized treatment planning, IMRT is time consuming and carries large technical requirements. Tomita et al. [16] concluded that IMRT provides a dosimetric advantage over 3DCRT in terms of PTV coverage, according to an analysis of radiotherapy treatment plans in NK/T-cell lymphoma cases. However, the clinical date comparing IMRT and 3DCRT was still lacking.

In this study, we compared the two widely used radiotherapeutic techniques of 3DCRT and IMRT for the treatment of NK/T-cell lymphoma using statistically valid methodologies. 3DCRT and IMRT were compared in terms of differences in PTV dose coverage and OAR sparing. Moreover, the clinical results of these two techniques were analyzed, particularly regarding LCP, OS, prognostic factors, and radiotherapy-related adverse events.

## Methods

This was a retrospective, single-center study. We made our sample selection from 119 continuous treated patients who had pathological diagnoses of extranodal NK-T cell lymphoma, nasal type at the institute between May 2005 and December 2008. Eligible patients needed to have previously untreated Ann Arbor stage I-II disease within the upper aerodigestive tract. This study sought to compare the treatment outcomes and toxicity levels between the 3DCRT and IMRT radiotherapy techniques. Patients with the following conditions were excluded: 1) previous head and neck irradiation exposure; 2) death from disease progress or adverse toxicity during or right after induction chemotherapy; and 3) declination to receive definitive radiotherapy for various reasons (mental disease or personal decision).

### Patient characteristics

Ninety-four patients were identified as fulfilling our inclusion criteria (Table 1). The median patient age was 44 years (range, 14–73). The male/female ratio was 2.4:1. Of these, 76 patients were in stage I and 18 patients were in stage II with positive cervical lymph nodes according to Ann Arbor staging. Most of the patients (85.1%) had primary disease in the nasal cavity, whereas the other patients had disease originating in the nasopharynx, oropharynx or tonsil. Forty-seven cases were treated with 3DCRT, and the others were treated with IMRT. Following the development of radiation techniques at our institution, the percentage of IMRT usage was increased significantly after 2007, from 19.3% between 2005 and 2007 to 97.3% during 2008.

**Table 1:** Patient characteristics

**Characteristic**	**3DCRT (n=47)**	**IMRT (n=47)**	***P *value**
	**No. of patients (%)**	**No. of patients (%)**	
Sex			0.37
Male	35(74.5)	31(66.0)	
Female	12(25.5)	16(34.0)	
Age, years			0.37
Median range	44(20–70)	43(14–73)	
≤60	42(89.4)	39(83.0)	
>60	5(10.6)	8(17.0)	
ECOG score			0.24
0	22(46.8)	27(57.4)	
1	20(42.6)	19(40.4)	
2	5(10.6)	1(2.1)	
Stage			0.60
IE	39(83.0)	37(78.7)	
IIE	8(17.0)	10(21.3)	
B symptoms			0.10
Absent	29(61.7)	21(44.7)	
Present	18(38.3)	26(55.3)	
LDH level			0.63
Normal	35(74.5)	37(78.7)	
Elevated	12(25.5)	10(21.3)	
Primary site			0.24
Unilatera nasal cavity limited	16(34.0)	11(23.4)	
Bilateral nasal cavity limited	5(10.6)	1(2.1)	
nasal cavity lesions with limited invasion ^a^	9(19.1)	15(31.9)	
nasal cavity lesions with extensive invasion^b^	10(21.3)	13(27.6)	
Non-nasal cavity originated^c^	7(14.9)	7(14.9)	
mIPI			0.30
0	23(48.9)	28(59.6)	
1	20(42.6)	13(27.6)	
2-3	4(8.5)	6(12.8)	
Induction chemotherapy			<0.01
None	10(21.3)	1(2.1)	
CEOP	27(57.4)	2(4.2)	
DICE	10(21.3)	44(93.6)	

^a^nasal cavity lesions with limited invasion: the tumor invaded nasal cavity and one neighboring anatomic structure (pnasal sinus or nasopharynx).

^b^nasal cavity lesions with extensive invasion: the tumor invaded nasal cavity and two or more neighboring anatomic structures(pnasal sinuses, nasopharynx, oropharynx or laryngopharynx).

^c^Non-nasal cavity originated: the tumor originated from nasopharynx, oropharynx, laryngopharynx or tonsil.

Abbreviations: *LDH* lactate dehydrogenase, *ECOG* Eastern Cooperative Oncology Group, *mIPI* Stage-modified International Prognostic Index, *CEOP* cyclophosphamide, epirubicin, vincristine prednisone, *DICE* etoposide. cyclophosphamide cisplatin, dexamethasone.

### Treatment

Of the 94 patients included, 11 were treated with definitive radiotherapy alone. The remaining 83 patients were treated with a combined treatment consisting of one to four cycles of induction chemotherapy and definitive radiotherapy, as shown in Table 1. The choice to administer induction chemotherapy was decided by the physicians. Those patients demonstrating high risk factors, such as stage II disease, B symptoms, or large extensions of primary lesions, generally underwent induction chemotherapy.

Two chemotherapy regimens were applied: CEOP (cyclophosphamide, epirubicin, vincristine, prednisone) for 29 patients and DICE (etoposide. cyclophosphamide cisplatin, dexamethasone) for 54 patients. Both treatments were repeated every 3 weeks. Considering the evidence demonstrating the relative resistance of NK/T-cell lymphomas to anthracycline-based chemotherapy regimens [4,5], CEOP regimens were gradually replaced by DICE regimens at our institution from Mar 2006.

### Radiotherapy

All patients were immobilized in a supine position with a thermoplastic head mask and simulated by CT using a 3 mm slice thickness from the vertex of the skull to inferior of the clavicular heads.

**Figure 1:**
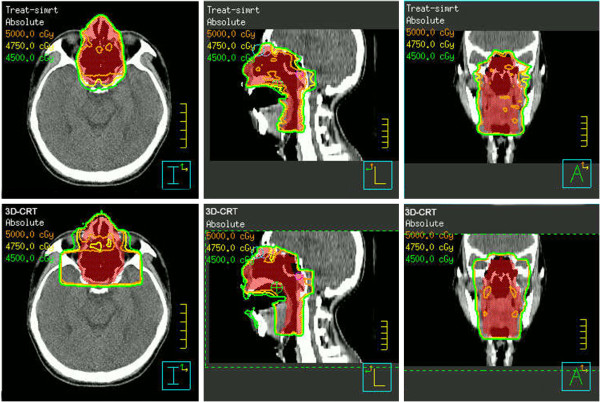
**Example of the dose distribution using 3DCRT and IMRT.** This patient had a tumor limited to the bilateral nasal cavity and nasopharynx and obtained CR following induction chemotherapy. The PTV (volume in red) included the nasal cavity, ethmoid sinus, maxillary sinus, nasopharynx and laryngopharynx. The top row and the bottom row show the dose distributions for IMRT and 3DCRT, respectively.

The gross tumor volume (GTV) was determined according to CT or MRI analysis and endoscopic findings. The clinical target volume (CTV) consisted of the GTV and adjacent structures with potential involvement. If the neck lymph nodes were involved, the ipsilateral neck was irradiated. No prophylactic cervical irradiation was delivered. The CTV was expanded by 5 mm to obtain the PTV, and one example of PTV delineation is shown in Figure 1.

The requirements for the treatment plans were as follows: 1) 100% of the PTV volume received at least 95% of the prescription dose and more than 95% of the PTV volume received a 100% prescription dose; and 2) less than 5% of the PTV volume received more than 110% of the prescribed dose. OARs included the brainstem, spinal cord, lenses, eyes, and parotid glands. According to published QUANTEC articles [17], the dose constrictions of the OARs were as follows: brainstem, maximum dose <50 Gy; spinal cord, maximum dose ≤45 Gy; lens, maximum dose 10 <Gy; parotid glands, mean dose <26 Gy.

A three-field 3DCRT technique using one anterior portal and two lateral fields with 6 MV was applied. In addition, an appropriate 9-MeV or 12-MeV electron beam was used to compensate the insufficiency dose of the anterior ethmoid. For IMRT planning, 5–7 fields were given. The prescription was for 50 Gy in 25 fractions at 2 Gy per day for 5 days per week.

### Comparison criteria for the radiation treatment plans

**Figure 2:**
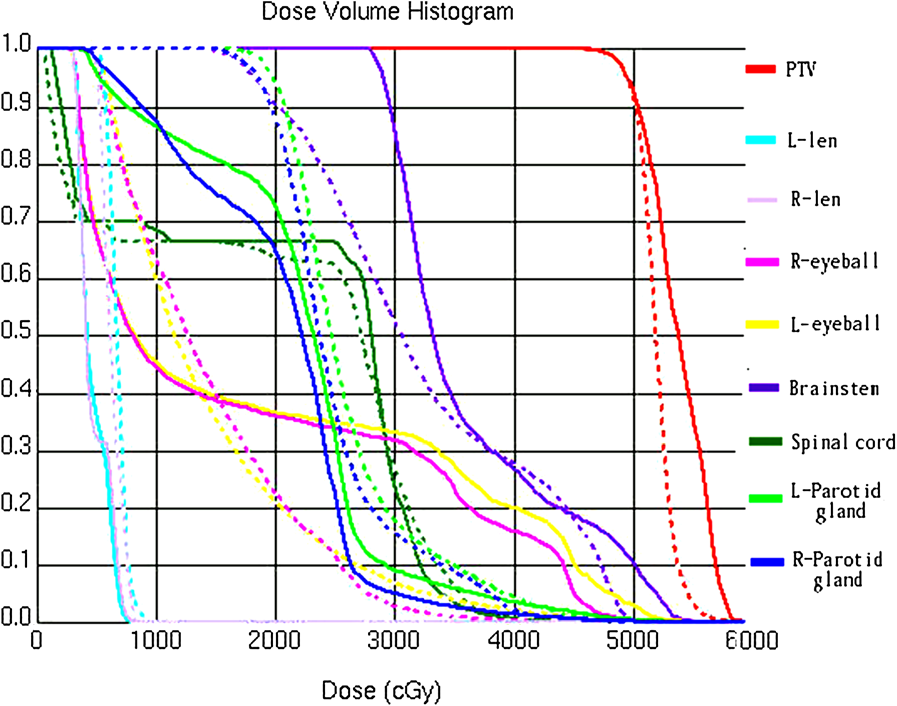
**DVH for the 3DCRT and IMRT plans for the same patient shown in Figure **1**.** The solid lines and the dash lines represent the DVH of 3DCRT and IMRT, respectively.

The data from the dose volume histograms (DVHs) obtained from each patient were analyzed. Representative dose distribution and DVHs for 3DCRT and IMRT are shown in Figures 1 and 2. The dose coverage was analyzed according to the minimum dose (D_min_), maximum dose (D_max_), mean dose (D_mean_), V_95%_ (i.e., the percent of the volume that receives x% of the prescription), V_90%_, V_105%_, V_110%_, conformity index (CI), and homogeneity index (HI). The CI was defined as follows [18]:

$$CI=\frac{V_{\mathrm{RI}}}{\mathrm{PTV}}$$

where V_RI_ represents the volume covered by the prescription dose (50 Gy). A CI value of 1.0 indicates that the volume of the prescription isodose surface is equal to that of the PTV. The HI was defined as follows [19]:

$$\begin{array}{l} \mathrm{HI}=\frac{D_{2}-D_{98}}{D_{50}} \end{array}$$

where D_x%_ represents the dose delivered to x% of the PTV. Lower HI values indicate a more homogeneous target dose. OARs (e.g., the parotid glands, lens, eyeballs, brainstem, and spinal cord) were compared according to the values of D_max_ and D_mean_.

### Follow-up

The median duration of follow-up was 46 months, and the median duration of follow-up for the surviving patients was 50.4 months. For the 3DCRT group and the IMRT group, the median duration of follow-up for the surviving patients was 59.6 (46.5-73.3) months and 40.9 (35.0-56.1) months, respectively. All patients were regularly followed until death or the last follow-up at 3- to 6-month intervals. Tumor responses were assessed one month after the completion of RT using image methods, including endoscopic examinations, CT or MRI scans of the head and neck, or positron emission tomography. According to the CTCAE 3.0 criteria, the clinicians estimated radiation-related adverse events such as oral mucositis, xerostomia, or decreased visual acuity.

### Statistical analysis

Tumor response to radiotherapy was evaluated according to the Revised Response Criteria of Malignant Lymphoma [20]. OS was defined as the date of diagnosis to either the date of death from any cause or the last follow-up visit. LCP was calculated according to the date of diagnosis until the date of in-field or marginal recurrence.

The comparison of pmeters between IMRT and 3DCRT was calibrated using an independent samples *t*-test. The Kaplan-Meier method was used to calculate the LCP and OS curves. The log-rank test was performed for treatment comparisons in the univariate analysis. Moreover, all variables with a *p* value of <0.05 in the univariate analyses, in addition to radiation technique (IMRT or 3DCRT), were included in the multivariate model using the Cox proportional hazards regression model. Two-sided *p* values <0.05 were considered significant.

## Results

### Treatment plan: dose coverage and OAR sparing

pmeters related to dose coverage planning for 3DCRT and IMRT are presented in Table 2. The results indicated that IMRT showed better PTV coverage. The V_95%_ for IMRT was 96.17±13.36%, whereas that for 3DCRT was 85.40±27.04% (*p*=0.02). However, this advantage for IMRT was not observed for the comparison of V_90%_. The hot dose volume of 105% with IMRT was larger than that for 3DCRT, whereas the V_110%_ value was smaller for IMRT. As dose coverage (V_90%_, V_95%_, V_105%_, V_110%_), there was no significant difference observed, which is independent of undergoing cervical lymph node irradiation or not. The IMRT plan was also less homogeneous than that for 3DCRT with marginal significance (HI for 3DCRT: 0.20±0.11 vs. HI for IMRT: 0.10±0.03 *p*=0.05), whereas there was no significant difference between the IMRT and 3DCRT plans in terms of dose conformity (CI: 1.28±0.18 for 3DCRT vs. 1.08±0.16 for IMRT, *p*=0.07).

**Table 2:** PTV dose coverage

	**3DCRT**	**IMRT**	***p *value**
D_max_(Gy)	59.25±6.71	57.18±2.17	0.05^*^
D_mean_(Gy)	51.73±1.01	52.06±1.14	0.14
V_95%_(%)	85.40±27.04	96.17±13.36	0.02^*^
V_90%_(%)	98.70±3.32	98.99±1.81	0.60
V_105%_(%)	28.53±19.09	44.44±21.11	<0.01^*^
V_110%_(%)	7.14±7.92	4.19±5.10	0.04
HI	0.20±0.11	0.10±0.03	0.05^*^
CI	1.28±0.18	1.08±0.16	0.07

^*^variable with significant difference.

*D*_*max*_ maximum dose, *D*_*mean*_ mean dose, *V*_*x%*_ percent of volume receives x% of the prescription, *CI* conformity index, *HI* homogeneity index.

Regarding the comparison of parotid D_mean_, there was no significant difference between 3DCRT and IMRT (D_mean:_ 22.12±9.30 Gy for 3DCRT vs. D_mean:_ 20.91±6.56 Gy for IMRT, *p*=0.31). The result of 18 patients receiving cervical lymph node irradiation concludes to a higher parotid D_mean_ comparing to the counterpart 76 patients not receiving the corresponding irradiation (D_mean:_ 31.49±7.09 Gy vs. 20.93±6.66 Gy, *p*=0.00). Among the patients receiving cervical irradiation, those in the IMRT group (10 patients) demonstrated lower parotid D_mean,_ values in comparison to those in the 3DCRT group (8 patients) (IMRT: 27.23±7.0 Gy vs. 3DCRT: 32.93±9.69 Gy, *P*<0.05). The average maximal dose to the brainstem, spinal cord and lens (3DCRT vs IMRT) was 45.4 vs 41.4, 39.3 vs 32.5, and 7.5 vs 5.7 Gy, respectively. The average mean dose to eyeball was 13.2 vs 8.9 Gy for 3DCRT and IMRT, respectively. Similar to the parotid glands, the dosimetric details for the spinal cord, brainstem, lens, and eyeball demonstrated no significant differences between the IMRT and 3DCRT plans and all of these organs were strictly maintained within the dose limitations.

### Treatment response

Dramatic responses were observed in both the 3DCRT and IMRT groups. Complete remission (CR) was achieved in 46 of 47 patients (97.9%) in the 3DCRT group and in 45 of 47 patients (95.7%) in the IMRT group after the completion of all treatments.

Moreover, all 11 patients (10 in the 3DCRT group and 1 in the IMRT group) who were treated with radiation alone achieved CR. The CR rate was 31.3% following induction chemotherapy for 83 patients treated with chemoradiantion, and it rose to 97.3% in 3DCRT group and 97.8% in IMRT group respectively after completion of radiotherapy.

In the 3DCRT group, 91.5% of the patients completed the radiotherapy treatment to the total dose of 50 Gy. Three patients stopped radiotherapy at a total dose of 44–48 Gy due to oral mucositis, and one patient stopped treatment due to a pulmonary infection at a total dose of 28 Gy. In the IMRT group, 93.6% of the patients completed the radiotherapy treatment. Three patients discontinued treatment due to systemic progression at total doses of 12 Gy, 38 Gy, and 46 Gy.

### Treatment outcome and prognostic factors

The 4-year OS and 4-year LCP rates were 81.1%, and 87.2% for all patients, respectively. Treatment failures occurred in 31 patients, including 23 cases of systemic recurrence, 3 cases of residual lesions and 9 cases of local relapse (8 in-field or marginal recurrence and 1 outside-field recurrence in the gingiva). No cervical failure was observed.

**Figure 3:**
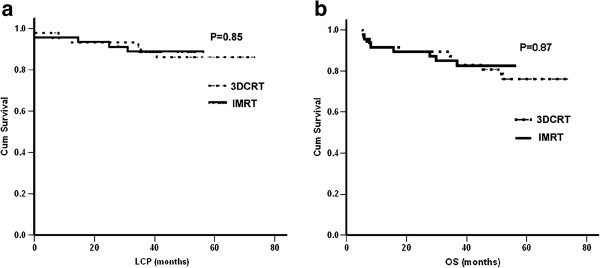
LCP (a) and OS (b) in patients treated with 3DCRT or IMRT.

All the variables (Sex, Male, Age, ECOG score, Stage, “B” symptoms, LDH level, Primary site, Stage-modified IPI, Induction chemotherapy, Response to induction chemotherapy, Radiotherapy) were under the univariate analysis (Additional file 1: Table e1). IMRT was not associated with improvement in terms of 4y-OS (80.9% for 3DCRT vs. 82.7% for IMRT, *p*=0.87) and 4y-LCP (86.3% for 3DCRT vs. 88.9% for IMRT, *p*=0.85) (Figure 3).

The significant factors identified in the univariate analysis (listed in Table 3), in addition to the radiotherapy technique (IMRT or 3DCRT), were also accounted for in the multivariate analysis. Independent poor prognostic factors for LCP included an ECOG score of 2 (hazard ratio: 2.23; 95% CI: 0.76-6.54; *P*=0.02), while an ECOG score of 2 (hazard ratio: 2.59; 95% CI: 1.08-6.22; *P*=0.03) and elevated LDH level (hazard ratio: 3.05; 95% CI: 1.03-9.06; *P*=0.04) were shown to be factors associated with poor OS. IMRT was also not found to be associated with improved LCP or OS.

**Table 3:** Multivariate analysis for LCP and OS

**Factor**	**OS**		**LCP**	
	**Hazard ratio**	***p *value**	**Hazard ratio**	***p *value**
	**(95% CI)**		**(95% CI)**	
IMRT	1.12(0.43–2.93)	0.81	1.05(0.31–3.59)	0.94
ECOG score =2	2.59(1.08–6.22)	0.03	2.23(0.76–6.54)	0.02
LDH elevated^¶^	3.05(1.03–9.06)	0.04	–	–
Stage = II^±^	–	–	2.10(0.29–15.00)	0.46
Nasal cavity lesions with extensive invasion^&^	1.30(0.83–2.06)	0.25	1.04(0.54–2.00)	0.92
Stage-modified IPI=2-3	0.95(0.40–2.24)	0.91	1.14(0.36–3.65)	0.83

^¶^LDH elevation was accounted for in the multivariate analysis for LCP, but not for OS.

^±^Stage = 2 was accounted for in the multivariate analysis for OS, but not for LCP.

^&^Nasal cavity lesions with extensive invasion: the tumor invaded nasal cavity and two or more neighboring anatomic structures(pnasal sinuses, nasopharynx, oropharynx or laryngopharynx).

### Toxicity related to radiotherapy

Oral mucositis was the most common type of toxicity, although only three patients ceased treatment due to grade IV oral mucositis (all in 3DCRT group). Following a long time follow-up, late toxicity was observed. In both 3DCRT group and IMRT group, 43/47 patients (91.5%) and 42/47 patients (89.4%) had toxicity data at 1 year and 2 years follow-up, respectively. There was no significantly different rate of xerostomia between the IMRT group and the 3DCRT group(12.8% Grade I; 6.4% Grade II for 3DCRT vs. 21.3% Grade I; 4.2% Grade II for IMRT, no higher grade xerostomia was observed). However, it was observed a lower xerostomia rate in patients not receiving cervical irradiation. In clinical follow-up, grade I and II xerostomia rate reached to 38.9% in cervical irradiation group while it was only 18.4% in no cervical irradiation group. Among those 18 cases who underwent cervical irradiation, IMRT group (10 patients) showed a lower rate of xerostomia (30% grade I cases and no serious cases), while 3DCRT group (8 patients) demonstrated a 38% grade I and 12% grade II xerostomia rate. One patient experienced hypogeusia in the IMRT group, whereas 1 patient in the 3DCRT group experienced hyposmia. No cataracts or nervous system disorders were observed during follow-up.

## Discussion

Both the 3DCRT and IMRT are widely used and each has some specific advantages. The IMRT shows superior dosimetric benefits while the 3DCRT is easy to implement even for those Linac without IMRT feasibility. The current study found that for cases of NK/T-cell lymphoma, IMRT demonstrated improved dose coverage but was equivalent to 3DCRT in terms of LCP, OS and toxicity. Furthermore, definitive radiotherapy with a regional field to a total dose of 50 Gy was shown to be efficient and safe for NK/T-cell lymphoma using either IMRT or 3DCRT. Tomita et al. [16] found that IMRT achieved significantly better dose coverage in terms of V90% and V95% compared to 3DCRT. Consistent with these findings, the current study observed improved V95% dose coverage with IMRT in NK/T-cell lymphoma(85.40±27.04% for 3DCRT vs. 96.17±13.36% for IMRT, *P*=0.02). However, the clinical results from this long-term follow-up study showed that the improved dose coverage of IMRT failed to provide consequently improved local control and overall survival, as IMRT was not associated with LCP and OS in both the univariate and multivariate analysis.

Salivary gland hypofunction and xerostomia have been shown to be common adverse events related to external radiotherapy in the head and neck region and can affect a patient’s oral health-related quality of life [21]. In our study, the parotid D_mean_ value was strictly limited within the constraint of 26 Gy for both IMRT and 3DCRT, which, in combination with the low total dose of 50 Gy delivered to cases of NK/T-cell lymphoma, may have contributed to the lack of parotid sparing associated with IMRT. We observed superior parotid avoidance in treatment planning of IMRT group among 18 patients who underwent cervical region irradiation. This result might be attributed to the neighboring PTV to parotid glands in cervical irradiation planning. It implied a higher parotid Dmean for the both plannings, and IMRT might take advantage of better OAR protection. However, it was difficult to analyze differences in the xerostomia rate between these two groups due to the small sample size of only 18 patients. Nonetheless, our results suggest that IMRT may reduce parotid gland hypofunction when the cervical region is included in the treatment volume, although this advantage should be more carefully examined in additional cases of cervical irradiation.

Although most reports do not clearly delineate the radiation field, a majority of researchers prefer the extended radiation field [2,4,7,10]. In addition, a total dose equal to or greater than 45–50 Gy was shown to achieve excellent local control in previous trials [7,11]. Based on these data, our institution designed a radiotherapy schedule with a prescribed dose of 50 Gy and the use of a regional field that encompassed the anatomic structure(s) involved as well as adjacent structures. Using this schedule, dramatic responses to radiotherapy were observed for patients in both the 3DCRT and IMRT groups. Of the patients who received combined therapy, the low CR rate after induction chemotherapy increased following the completion of definitive radiotherapy. Moreover, adequate local control and OS were achieved. These results suggested that radiotherapy performed according to this RT schedule provided good treatment outcomes for patients with NK/T-cell lymphoma, regardless of whether IMRT or 3DCRT was applied. Moreover, OARs were strictly kept within the safe dose range for both treatment groups, and no severe toxicity was observed at subsequent follow-up visits. Therefore, 3DCRT was shown to be as safe as IMRT using this radiotherapy schedule for NK/T cell lymphoma.

Together, these results suggest that IMRT failed to demonstrate any advantages over IMRT for the treatment of NK/T cell lymphoma. In addition, these techniques were equivalent in terms of both local control and reducing the rate xerostomia, which is inconsistent with previous results in patients with head and neck cancer [12]. High sensitivity to radiation and a lower prescribed dose for lymphoma treatment likely made any advantages of IMRT difficult to detect. Considering its easier treatment planning and time-sparing, 3DCRT could be recommended for most cases of NK/T-cell lymphoma. Satisfying levels of local control were achieved using definitive radiotherapy in some recent studies, including ours [4,5]. However, the relatively high distant failure rate of 24.5% in the current study represents one remaining obstacle related to this type of treatment and highlights the need to improve systemic treatment options. Moreover, chemotherapy regimens that do not contain anthracycline have demonstrated advantages in recent studies [22,23]. Although the DICE regimen was not found to be significant factor associated with improved OS in the current study, this relationship will be further studied in prospective, randomized clinical trials at our institution.

## Conclusions

This research demonstrated that radiotherapy using a regional field to a total dose of 50 Gy was effective and safe, using either 3DCRT or IMRT, for patients with stage I-II NK/T-cell lymphoma. IMRT demonstrated the advantage of better dose coverage, although this was not associated with improvements in LCP or OS. Furthermore, IMRT may have potential of reducing parotid gland hypofuction in cases where the cervical region is included in the treatment volume. In further study, chemotherapy regimens should be more carefully investigated with the goal of improving systemic treatment.

## Abbreviations

3DCRT: Three-dimensional conformal radiotherapy; IMRT: Intensity-modulated radiotherapy; LCP: Local control probability; OS: Overall survival; CR: Complete remission; Dmean: The mean dose; Dmin: The minimum dose; Dmax: The maximum dose; GTV: Gross tumor volume; CTV: Clinical target volume; PTV: Planning treatment volume; MRI: Magnetic resonance imaging; DVHs: Dose volume histograms; OAR: Organs at risk.

## Competing interests

The authors declare that they have no competing interests.

## Authors' contributions

QS and XM carried out the the review of the patients, performed the statistical analysis and drafted the manuscript. WH, LC, JH participated in the analysis of the treatment of radiotherapy plans. YG participated in its design and coordination and helped to draft the manuscript. All authors read and approved the final manuscript.

## Supplementary Material

Additional file 1 Univariate analysis for LCP and OS. Click here for file
