# Supplementary material for: Intensity-modulated radiotherapy versus three-dimensional conformal radiotherapy for stage I-II natural killer/T-cell lymphoma nasal type: dosimetric and clinical results
Source: Radiat Oncol. 2013 Jun 25;8:152. doi: 10.1186/1748-717X-8-152 (PMC3723914; doi:10.1186/1748-717X-8-152)
Supplement: Additional file 1 — Univariate analysis for LCP and OS. [file 1748-717X-8-152-S1.doc]

Table e1 Univariate analysis for LCP and OS

| Prognostic Factor | 4-Year OS | | 4-Year LCP | |
| --- | --- | --- | --- | --- |
|  | % | *p* value | % | *p* value |
| Sex |  | 0.38 |  | 0.06 |
| Male | 84.0 |  | 91.9 |  |
| Female | 74.3 |  | 75.3 |  |
| Age, years |  | 0.78 |  | 0.20 |
| ≤60 | 78.3 |  | 85.4 |  |
| >60 | 83.3 |  | 100 |  |
| ECOG score |  | <0.01^*^ |  | <0.05^*^ |
| 0 | 91.6 |  | 90.8 |  |
| 1 | 72.9 |  | 88.3 |  |
| 2 | 50.0 |  | 50.0 |  |
| Stage |  | 0.41 |  | 0.02^*^ |
| IE | 82.6 |  | 91.3 |  |
| IIE | 74.1 |  | 71.1 |  |
| “B” symptoms |  | 0.53 |  | 0.23 |
| Absent | 81.9 |  | 90.9 |  |
| Present | 79.2 |  | 83.3 |  |
| LDH level |  | <0.01^*^ |  | 0.20 |
| Normal | 87.2 |  | 89.4 |  |
| Elevated | 62.0 |  | 79.2 |  |
| Primary site |  | <0.05^*^ |  | <0.05^*^ |
| Nasal cavity lesion with and without limited invasion ^a^ | 87.6 |  | 92.2 |  |
| Nasal cavity lesions  with extensive invasion ^b^ | 67.2 |  | 76.2 |  |
| Non-nasal cavity ^c^ | 77.9 |  | 84.4 |  |
| Stage-modified IPI |  | <0.05^*^ |  | <0.05^*^ |
| 0 | 89.6 |  | 91.0 |  |
| 1 | 74.2 |  | 86.8 |  |
| 2 - 3 | 60.0 |  | 65.6 |  |
| induction chemotherapy |  | 0.58 |  | 0.24 |
| Absent | 72.7 |  | 100.0 |  |
| Present | 82.0 |  | 85.7 |  |
| Chemotherapy regimen |  | 0.88 |  | 0.50 |
| CHOP | 79.3 |  | 88.8 |  |
| DICE | 85.0 |  | 84.6 |  |
| Response to induction chemotherapy |  | 0.63 |  | 0.76 |
| CR | 84.4 | - | 82.4 |  |
| Non-CR | 81.3 | - | 87.1 |  |
| Radiotherapy |  | 0.87 |  | 0.85 |
| 3DCRT | 80.9 |  | 86.3 |  |
| IMRT | 82.7 |  | 88.9 |  |

^*^variable with significant difference.

Abbreviations: LDH, lactate dehydrogenase; ECOG, Eastern Cooperative Oncology Group; mIPI, Stage-modified International Prognostic Index; CEOP, cyclophosphamide, epirubicin, vincristine prednisone; DICE, etoposide. cyclophosphamide cisplatin, dexamethasone.

^a^ nasal cavity lesion with and without limited invasion: the tumor invaded nasal cavity only or invaded nasal cavity and one neighboring anatomic structure (paranasal sinus or nasopharynx).

^b^nasal cavity lesions with extensive invasion: the tumor invaded nasal cavity and two or more neighboring anatomic structures(paranasal sinuses, nasopharynx, oropharynx or laryngopharynx).

^c^non-nasal cavity originated: the tumor originated from nasopharynx, oropharynx, laryngopharynx or tonsil.
